# The Arthroscopic Ulnohumeral Arthroplasty: From Mini-Open to Arthroscopic Surgery

**DOI:** 10.1155/2011/798084

**Published:** 2011-06-30

**Authors:** Ilse Degreef, Luc De Smet

**Affiliations:** Department of Orthopaedics, University Hospitals Leuven, Pellenberg Campus, Weligerveld 1, 3212 Pellenberg, Belgium

## Abstract

In cubarthritis—osteoarthritis of the elbow—surgical procedures may be considered to debride the elbow joint to reduce pain, to increase mobility, and to postpone joint replacement surgery. The ulnohumeral arthroplasty as described by Outerbridge and Kashiwagi was originally introduced to debride both anterior and posterior elbow compartments through a direct posterior mini-open approach. To achieve this, a distal humeral fenestration throughout the humeral fossa is performed. Although with an elbow arthroscopy, a technique that was obviously developed later on, all compartments can be easily visualized. The arthroscopic fenestration of the humerus preserves its advantages, with good clinical results focused on pain relief and gaining mobility. On top, future elbow joint locking based on degenerative loose bodies can be prevented. Therefore, this surgery is often done in young, more active patients and even in sportsmen. These patients, however, need to be prompted to restrict loading on the elbow in the immediate postoperative period, because the elbow is biomechanically weakened and may be prone to a fracture. However, both outcome and postoperative rehabilitation are promising and the arthroscopic Outerbridge procedure is a reliable procedure with an easy rehabilitation. Therefore, the threshold is relatively low in early cubarthritis and recurrent locking of the elbow. In this paper, we present a literature review and the author's experience and own research on the Outerbridge procedure.

## 1. Introduction

Although relatively uncommon, osteoarthritis of the elbow or cubarthritis causes pain and mobility loss in the joint. An elbow impairment often results in a severe disability of the entire upper limb. A total elbow arthroplasty may be considered in severe elbow arthritis, certainly in the elderly. However, in mild cubarthritis and in younger patients, the decision for joint replacement may be harder to make because of risks of loosening on the long term. In these cases, the ulnohumeral arthroplasty or Outerbridge-Kashiwagi procedure is a reasonable alternative [1]. In this surgical procedure, a distal humeral fenestration through the olecranon fossa is done to achieve pain relief, reduce locking, and improve mobility of the elbow joint. Originally developed as a mini-open technique to perform a thorough debridement of all elbow compartments by a mini-open posterior approach, the technique is now also successfully used in arthroscopic procedures [2]. 

## 2. Material and Methods

A literature search was done on the history and outcome of both open and arthroscopic ulnohumeral elbow arthroplasty and Outerbridge-Kashiwagi procedure emphasizing the technique, results, and risks or complications. Search engines PubMed and MedLine were used. The experience of the author was added and compared to the literature.

## 3. History

The Outerbridge-Kashiwagi procedure was first introduced by Outerbridge and popularized by Kashiwagi in 1978 to treat mild to moderate cubarthritis [3]. In this degenerative elbow condition, osteophytes form on the olecranon, coronoid, and in their concomitant fossae in the distal humerus [4]. These osteophytes impinge on each other, which then limits the hinging elbow motion and causes pain. To address this problem, Kashiwagi developed the technique of distal humeral fenestration through a direct and limited posterior approach to remove loose bodies and osteophytes in both the anterior and posterior compartments. Morrey modified the technique with a triceps-sparing approach in 1993 [5].

Elbow arthroscopy was first attempted on a cadaver in 1931 by Burman [6]. He claimed the procedure was “unsafe,” due to the proximity of the ulnar, median and radial nerves and the brachial artery. It wasn't until 1980 that Ito introduced safe portals [1]. Since then, elbow arthroscopy increasingly gained importance and its effectiveness has improved for a wide variety of conditions. It is now used for the diagnosis of instability, removal of loose bodies, synovectomy, avascular necrosis, plica synovialis impingement, tennis elbow, radial head resection or osteosynthesis, capsulectomy in arthrofibrosis, and debridement of early cubarthritis [7, 8]. Redden and Stanley were the first to report satisfactory results with the arthroscopic Outerbridge-Kashiwagi procedure in 1993 [2]. 

## 4. Mini-Open Ulnohumeral Arthroplasty

In the open technique, the elbow joint is opened through a small posterior incision from the olecranon tip upwards over 4 to 6 centimeter. To do this, the patient is installed in lateral decubitus with the arm resting on a Mayo support with a 300 mmHg tourniquet. A direct posterior triceps splitting approach is used to open up the posterior elbow compartment. Then, using a 4 mm burr, the olecranon fossa is perforated. The hole is then enlarged with Kerrison Rongeurs to a width of 10 to 15 mm. Loose bodies are extracted, osteophytes on the olecranon tip are removed, and an anterior debridement is performed through the created distal humeral hole with a capsular release if necessary. Immediate active rehabilitation is encouraged.

## 5. Arthroscopic Procedure

The arthroscopic procedure is done in a lateral decubitus, the arm resting on a Mayo support with a 300 mmHG tourniquet. A 4.0 mm 30° arthroscope with a nonvented cannula is used for visualization. Through a proximal medial and a mid lateral portal, the anterior compartment is first debrided. Then, a direct posterior approach is done for the debridement, combined with a posterolateral approach for visualization. A 4 mm arthroscopic burr is used to perforate the distal humerus, ensuring that this is done in the middle of the distal humeral fossa with a 90° angle on the humerus. Arthroscopic portals are left open for easy relieve of swelling. A compressing bandage is replaced with small band aids after 5 days, and active rehabilitation is encouraged.

## 6. Biomechanics

**Figure 1:**
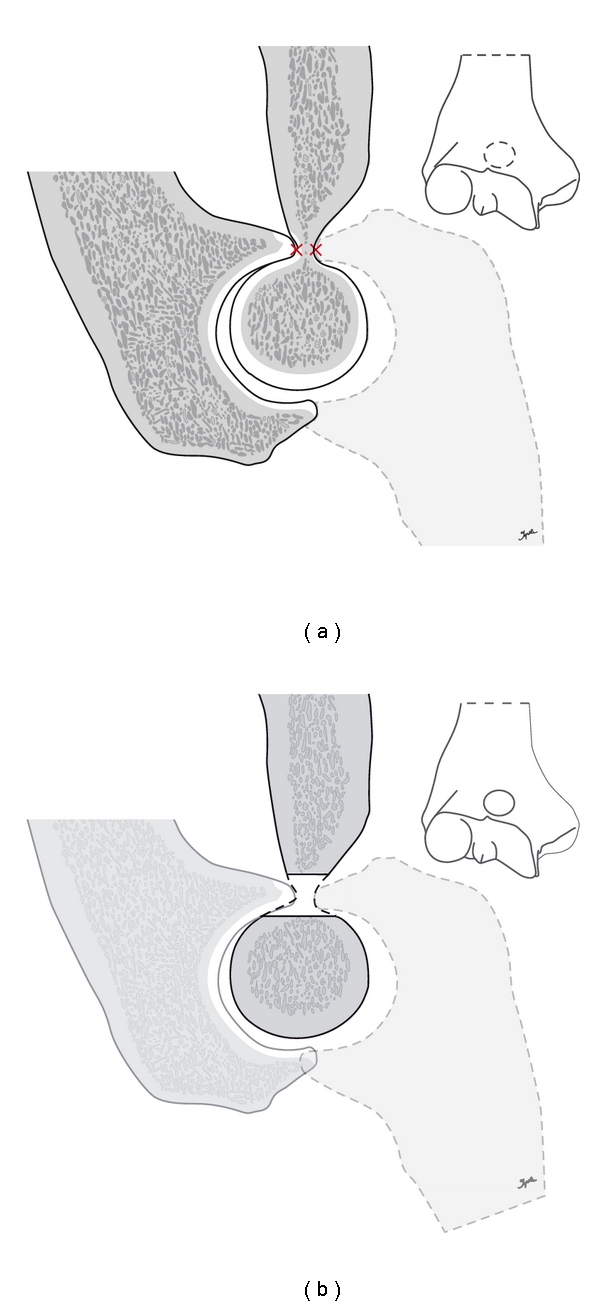
Schematic drawing of the impingement of the coronoid process and the olecranon tip in the anterior and posterior humeral fossae in case of early cubarthritis with the formation of osteophytes which impinge in maximal flexion and extension of the joint (a). This is resolved by a decompressing effect after trepanation of the distal humerus (b).

**Figure 2:**
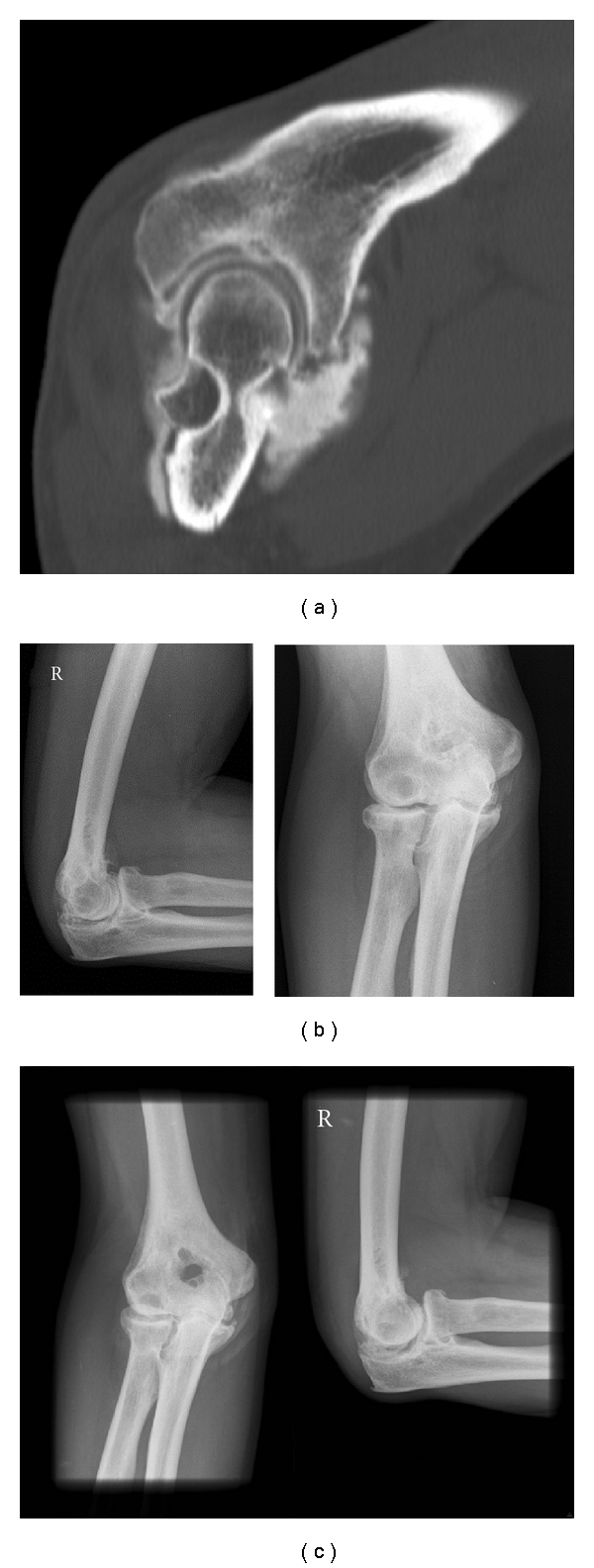
Radiological assessment with CT scan of early cubarthritis shows the posterior impingement in extension (a). Pre- (b) and postoperative (c) X-rays of the perforation of the distal humerus.

**Figure 3:**
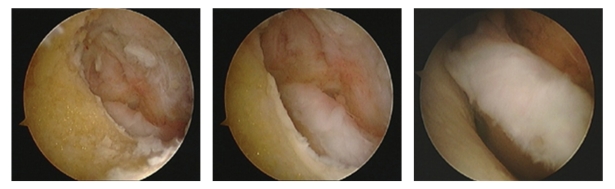
Intraoperative images of the perforated humerus (seen from the posterior compartment with a view on the anterior compartment of the joint) demonstrating the free movement of the coronoid process in the created hole.

Originally, the open procedure was introduced to approach both the anterior and the posterior compartments through a small posterior dissection. In arthroscopy, all compartments are easily addressed without perforating the distal humerus. In mild cubarthritis, a thorough arthroscopic elbow debridement with resection of loose bodies, synovitis, and osteophytes can improve complaints [7]. However, next to the joint debridement, an arthroscopic distal humeral fenestration may be associated, even though it is not strictly necessary for visualization (as was initially intended in the open procedure). In addition to improving joint visualization, the distal humeral fenestration also significantly reduces locking and impingement, leading to pain relief with an even easier rehabilitation with an arthroscopic technique. The clinical benefit is most likely due to the dynamic decompressing effect of the anterior and posterior elbow compartments in full flexion and extension (Figure 1). This decompression is achieved by the perforation of the distal humerus in the olecranon and coronoid fossa (Figure 2). As a result, remaining osteofytes on the olecranon tip and the coronoid processus run free in the created hole (Figure 3). 

Because of the easy rehabilitation and the low impact on the patient, the threshold is low and the arthroscopic Outerbridge-Kashiwagi is now more and more used in young patients, even in sportsmen [9]. This brings up the question as to what level the humerus is thus put at risk in their high-demand population. In a former study, we looked at the fracture risks in cadaver specimens [10]. We concluded that the fracture lines are displaced from humeral shaft fractures towards intraarticular column fractures in standard posterior forces on the distal humerus [11]. The columns also appear to be about 40% easier to break. In a clinical setting, bone remodeling is obvious after 6 weeks on radiological examinations, and although the hole remains visual, the columns are surrounded by cortical bone and the fracture risk most likely has disappeared. However, in the immediate postoperative period, patients need to be prompted to reduce sports activities, since bone strength of the distal humerus does not guarantee such a high reserve if maximal muscle forces are produced [2, 12].

## 7. Results

Minami reported good results with the open procedure reporting initial satisfactory results in over 90% in 1985 [4]. After a longer followup (9–16 y), however, these results decreased to 55% in 44 cases in 1996. These findings demonstrate a temporary result in most cases and this is confirmed by many reports. Although in short-term studies good to excellent outcomes are reported, long-term follow-up studies demonstrate a limited recurrence of the complaints, since obviously the underlying disease remains present. Relatively, short-term results after about 2 to 5 years as reported by Morrey in 1992 showed similar results of 80% success rate in 15 elbows, 81% in 36 elbows by Forster in 2001, 74% in 46 elbows by Antuna in 2002, 88% in 17 elbows by Sarris in 2004, and 87% in 16 elbows by our group in 2004 [5, 13–16]. The Mayo Performance Index improved from 63 to 88 and range of motion from 94° to 114°. However, in 2003 Philips presented a good outcome even after a longer followup with a minimum of 5 years with still a 85% success rate in 20 elbows [17], although results deteriorated somewhat after a longer followup, and surgical benefits were maintained in 80%.

Complications are uncommon in elbow arthroscopy with an incidence of less than 0.8% serious complications like joint infection and up to 11% minor complications like prolonged wound drainage, residual extension loss, or transient nerve palsy. The incidence of these complications is directly related to the surgeons experience in elbow arthroscopy [7, 18]. Antuna mentioned a risk for transient ulnar nerve paresthesia due to elongation if severe contractures were corrected [13]. Forster et al. mentioned ulnar nerve entrapment, a wound hematoma, a superficial infection, and a myocardial infarction [14]. Allen reported a supracondylar fracture that required open reduction and internal fixation [19]. Chandrasenan described an important heterotopic ossification in the triceps muscle after an open procedure, requiring surgical removal [20]. Although clinically insignificant, ectopic ossifications were also seen in some cases after an arthroscopic procedure [8, 15]. In our series, no complications were seen in the arthroscopic procedure, compared to a wound infection and a shoulder-hand syndrome in the open technique [8, 11].

With the arthroscopic procedure, first satisfactory results were reported in 1993 by Redden and Stanley [2]. Later on in 1995, O'Driscoll recommended arthroscopy to treat milder cases of osteoarthritis, reserving open debridement for more advanced cases [7]. In 1999, Savoie and Nunley reported overall good to excellent results in pain control and improved motion in a series of 24 patients, of whom 75% underwent an additional radial head resection [17]. Krishnan et al. reported good to excellent results in younger patients under fifty in 2007 (11 elbows), which somewhat extended the indications for the procedure [21]. This growing indication for the arthroscopic Outerbridge-Kashiwagi procedure was illustrated by our group in 2009 when we reported on the procedure in young sportsmen, and in 2010, showing good results in 85% of 20 elbows [9, 22]. Mayo Performance Index improved from 54 to 88 and range of motion from 94° to 123°. Compared to our earlier results after the (mini-) open procedure, these results show no disadvantage of the arthroscopic procedure. Rehabilitation is easier, faster and clinical results are comparable if pain, satisfaction, and motion gain are considered.

In 2000, Cohen et al. also compared his results of open (18 elbows) and arthroscopic procedure (26 elbows) [23]. He reported an increased range of motion of 8° and an improved pain score with 29% after arthroscopy in all elbows. In the open procedure, mobility improved with 19° and pain with 20%, with no improvement in 17%. The author concluded that mobility improved more after the open procedure, possibly due to a more extensive debridement of the posterior compartment. However, even though both procedures are effective, Cohen et al. reported better results in the arthroscopic procedure due to a more significant pain relief [23]. Since the rehabilitation after arthroscopy is easy and fast with few scar tissue, complication rates are very low and visualization of all compartments is more clear once the surgeon has built up sufficient experience. At our department, the open technique is considered in difficult cases with earlier surgery, in which neurovascular structures would be at high risk for arthroscopy, due to scar tissue formation.

## 8. Conclusion

Although originally intended for a better visualization of all compartments of the elbow joints with a mini-open approach, the Outerbridge-Kashiwagi procedure is now successfully used in arthroscopic techniques. The decompressing effect of the distal humeral fenestration gives pain relief, improves mobility, and avoids elbow locking. However, since the threshold for this surgical procedure is now low and it is also performed in the young and active population, the elbow may be at risk for intraarticular fractures in maximal loading immediately after surgery and some caution for resuming sport activities should be prompted.

## References

[1] Ito K (1980). Arthroscopy of the elbow joint. Cadaverstudy. *Arthroscopy*.

[2] Redden JF, Stanley D (1993). Arthroscopic fenestration for osteoarthritis. *Arthroscopy*.

[3] Kashiwagi D, Kashiwagi D (1985). Osteoarthritis of the elbow joint. Intra-articular changes and the special operative procedure, Outerbridge-Kashiwagi method. *Elbow Joint*.

[4] Minami M, Kato S, Kashiwagi D (1996). Outerbridge-Kashiwagi’s method for arthroplasty of osteoarthritis of the elbow: 44 elbows followed for 8-16 years. *Journal of Orthopaedic Science*.

[5] Morrey BF (1992). Primary degenerative arthritis of the elbow. Treatment by ulnohumeral arthroplasty. *Journal of Bone and Joint Surgery—Series B*.

[6] Burman MS (1931). Arthroscopy of the direct visualization of joints. An experimental cadaver study. *Journal of Bone and Joint Surgery—Series A*.

[7] O’Driscoll SW (1995). Arthroscopic treatment for osteoarthritis of the elbow. *Orthopedic Clinics of North America*.

[8] Savoie FH, Nunley PD, Field LD (1999). Arthroscopic management of the arthritic elbow: indications, technique, and results. *Journal of Shoulder and Elbow Surgery*.

[9] Degreef I (2009). Arthroscopic fenestration of the distal humerus: a viable option for painful elbow impingement in sportsmen. *Acta Orthopaedica Belgica*.

[10] Degreef I, Van Audekercke R, Boogmans T, De Smet L (2011). A biomechanical study on fracture risks in ulnohumeral arthroplasty. *Chirurgie de la Main*.

[11] Degreef I The arthroscopic outerbridge procedure in elbow surgery: technique, new indications and safety of the procedure.

[12] Amis AA, Dowson D, Wright V (1980). Analysis of elbow forces due to high-speed forearm movements. *Journal of Biomechanics*.

[13] Antuña SA, Morrey BF, Adams RA, O’Driscoll SW (2002). Ulnohumeral arthroplasty for primary degenerative arthritis of the elbow: long-term outcome and complications. *Journal of Bone and Joint Surgery—Series A*.

[14] Forster MC, Clark DI, Lunn PG (2001). Elbow osteoarthritis: prognostic indicators in ulnohumeral debridement—The Outerbridge-Kashiwagi procedure. *Journal of Shoulder and Elbow Surgery*.

[15] Sarris I, Riano FA, Goebel F, Goitz RJ, Sotereanos DG (2004). Ulnohumeral arthroplasty: results in primary degenerative arthritis of the elbow. *Clinical Orthopaedics and Related Research*.

[16] Vingerhoeds B, Degreef I, De Smet L (2004). Debridement arthroplasty for osteoarthritis of the elbow (Outerbridge-Kashiwagi procedure). *Acta Orthopaedica Belgica*.

[17] Phillips NJ, Ali A, Stanley D (2003). Treatment of primary degenerative arthritis of the elbow by ulnohumeral arthroplasty. A long-term follow-up. *Journal of Bone and Joint Surgery—Series B*.

[18] Savoie FH (2007). Guidelines to becoming an expert elbow arthroscopist. *Arthroscopy*.

[19] Allen DM, Devries JP, Nunley JA (2004). Ulnohumeral arthroplasty. *Iowa Orthopaedic Journal*.

[20] Chandrasenan J, Dias R, Lunn PG (2008). Heterotopic ossification after the Outerbridge-Kashiwagi procedure in the elbow. *Journal of Shoulder and Elbow Surgery*.

[21] Krishnan SG, Harkins DC, Pennington SD, Harrison DK, Burkhead WZ (2007). Arthroscopic ulnohumeral arthroplasty for degenerative arthritis of the elbow in patients under fifty years of age. *Journal of Shoulder and Elbow Surgery*.

[22] Degreef I, Samorjai N, De Smet L (2010). The Outerbridge-Kashiwaghi procedure in elbow arthroscopy. *Acta Orthopaedica Belgica*.

[23] Cohen AP, Redden JF, Stanley D (2000). Treatment of osteoarthritis of the elbow: a comparison of open and arthroscopic debridement. *Arthroscopy*.

